# Measurement of Primary and Secondary Stability of Dental Implants by Resonance Frequency Analysis Method in Mandible

**DOI:** 10.1155/2013/506968

**Published:** 2013-05-13

**Authors:** Mehran Shokri, Arash Daraeighadikolaei

**Affiliations:** ^1^Advanced Implant Surgery Private Practice, Iran; ^2^Restorative Department, Ahvaz Jundishapur Dental School, Ahvaz, Iran

## Abstract

*Background.* There is no doubt that the success of the dental implants depends on the stability. The aim of this work was to measure the stability of dental implants prior to loading the implants, using a resonance frequency analysis (RFA) by Osstell mentor device. *Methods.* Ten healthy and nonsmoker patients over 40 years of age with at least six months of complete or partial edentulous mouth received screw-type dental implants by a 1-stage procedure. RFA measurements were obtained at surgery and 1, 2, 3, 4, 5, 7, and 11 weeks after the implant surgery. *Results.* Among fifteen implants, the lowest mean stability measurement was for the 4th week after surgery in all bone types. At placement, the mean ISQ obtained with the magnetic device was 77.2 with 95% confidence interval (CI) = 2.49, and then it decreased until the 4th week to 72.13 (95% CI = 2.88), and at the last measurement, the mean implant stability significantly (*P* value <0.05) increased and recorded higher values to 75.6 (95% CI = 1.88), at the 11th week. *Conclusions.* The results may be indicative of a period of time when loading might be disadvantageous prior to the 4th week following implant placement. These suggestions need to be further assessed through future studies.

## 1. Introduction

Since more than a decade, resonance frequency analysis (RFA) has been used as a noninvasive, reliable, easily predictable, and objective method of quantifying implant stability [1, 2]. RFA has been widely used to determine the effects of immediate or early loading or assess changes in stability over time [3, 4]. However, the literature on the alterations of stability during the postplacement period still lacks sufficient evidence, and more studies on different systems and variables are needed. The aim of this study was to investigate the primary and the secondary stability of ITI implants using a RFA device to detect changes in stability during early healing following implant placement and to determine whether the implant stability quotient (ISQ) could predict proper loading time.

## 2. Materials and Methods

### 2.1. Patients

Included in the present prospective cohort study were patients over 40 years of age with at least six months of complete or partial edentulous mouth. Other inclusion criteria which were dependent on further clinical and paraclinical examinations included a bone height of equal to or more than 12 mm, a crest width of equal or higher than 6 mm, and a bone density of D2 or D3 as classified by Friberg et al. [3].

Excluded were the patients with systemically compromised conditions, for example, diabetes, osteoporosis, hypertension, cardiac problems or those with psychological disorders, advanced periodontal problems, poor oral hygiene, lack of cooperation, occlusal discrepancies, insufficient density or height of residual ridge, a history of radiotherapy, smoking, or par functional habits. 

### 2.2. Ethical Considerations

Our local board of research methodology and ethics peer reviewed and approved the study protocol. The junior author informed all candidates of the study procedure and obtained signed informed consents from all the included patients in advance.

### 2.3. Implants

The senior author selected all the implants based on the clinical and radiological examinations and performed all the surgeries, and the junior author assisted the Oral and Maxillofacial Surgeon with surgical procedures. Threaded SLA-coated ITI implants were used.

### 2.4. Surgery

NewTom VGI (NewTom VGI, QR Verona, Italy) cone beam computed tomography imaging device (Figure 3(c)) and Panoramic X-ray (Figures 3(b) and 4(a)) was used for preoperative planning. The study followed a one-stage surgical protocol (Figure 4(c)). Residual alveolar crest width as well as jawbone density was examined. Bone density was later confirmed intraoperatively by pilot drill. Before surgery, oral cavity was rinsed with chlorhexidine 0.2% (Shahrdarou, Tehran, Iran) for a minute. Antiinflammation therapy consisting of Novafen (400 mg Brufen + Acetaminophen 325 mg + Caffeine 40 mg) (Alhavi, Tehran, Iran) and antibiotic therapy consisting of Amoxicillin, Cefalexin, or Clindamycin (Tehran Chemie, Tehran, Iran) 1-2 g half an hour before surgery were performed orally. After the administration of sufficient local anesthesia (Llidocaine 2% with epinephrine; Daroupakhsh, Tehran, Iran) to the surgical site, the senior author made a midcrestal incision with two vertical releasing incisions, reflected full-thickness buccal and palatal mucoperiosteal flaps, and flattened the implantation bony surface. Implant sites were drilled (Straumann, Basel, Switzerland) with a speed from 400 to 600 rpm using intermittent motions without additional pressure, under copious saline irrigation. Implants were placed with an insertion torque of 35 N/cm. The healing screws were then secured to the fixtures (Figures 3(a) and 4(b)). Primary wound closure was achieved by placing single suture with silk 3-0 or 4-0 (Supasil, Tehran, Iran) that were removed after 7–10 days (Figure 4(c)).

### 2.5. Resonance Frequency Measurements

**Figure 1:**
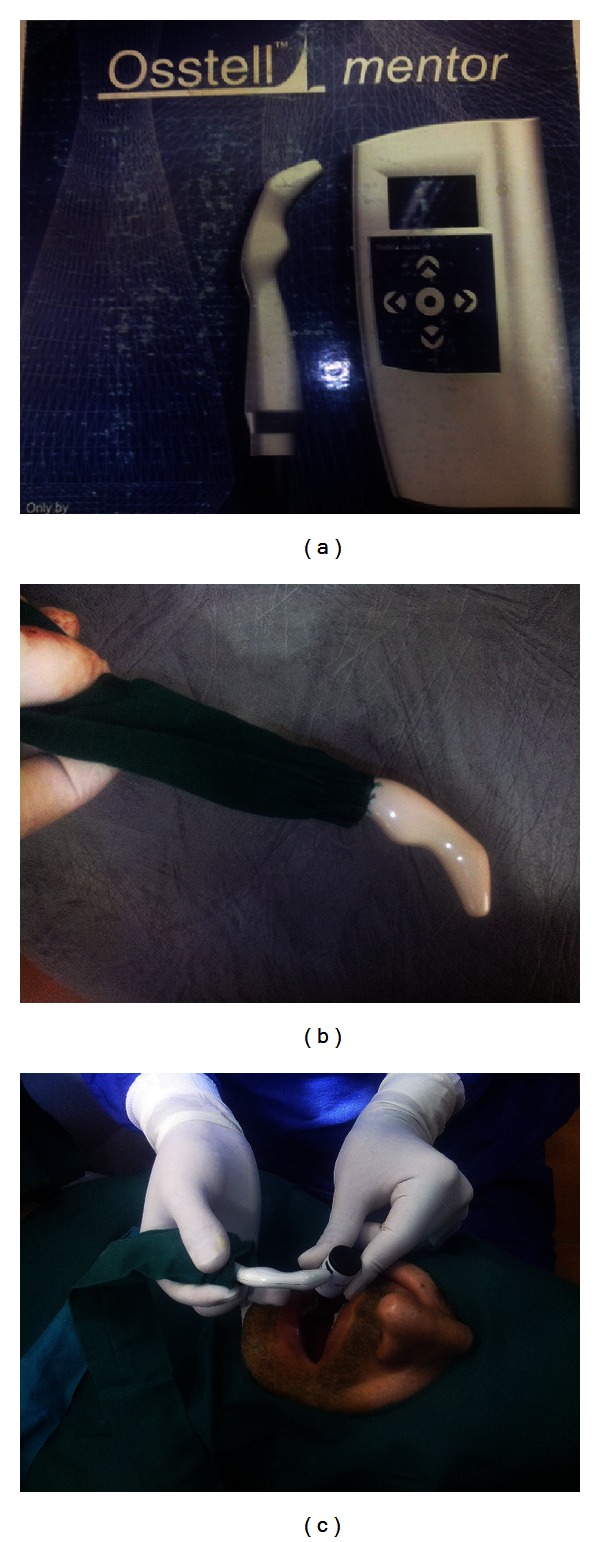
(a) Osstell mentor device. (b) Osstell mentor probe. (c) Osstell mentor device is in contact with the fixture immediately after placement to measure the baseline stability value of the implant. The same procedure was done once a week until the 5th week after surgery, and then was done once in the 7th and once in the 11th weeks.

Primary stability was measured using an Osstell mentor device (Figure 1), Integration Diagnostics, Savadaled, Sweden). All measurements were performed by the junior author, immediately after implant placement and weekly until week 5 and then at the 7th and 11th weeks. ISQ values were recorded into charts. A primary ISQ of 47 or less was considered a sign of questionable stability. The first two equal values were accepted as the authentic value. The authors followed the patients for 11 weeks after surgery during which they were not allowed to wear any provisional prostheses or insert any load to the fixtures (Table 1).

### 2.6. Statistics

Patients entered the study by sequential sampling method. The authors used Microsoft Excel 2007 to organize the data and assessed the data for possible statistical significance (*P* < 0.05) using paired *t* test.

## 3. Results

**Figure 2:**
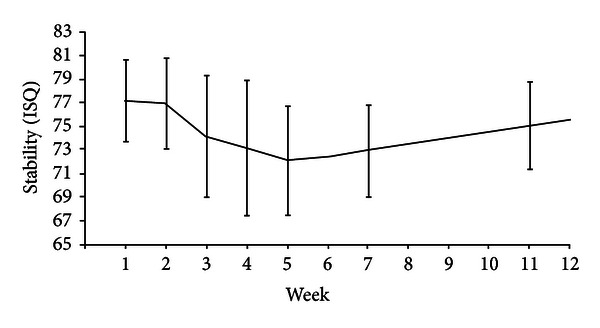
Illustration of the alternations of the mean implant stability values using resonance frequency analysis.

**Figure 3:**
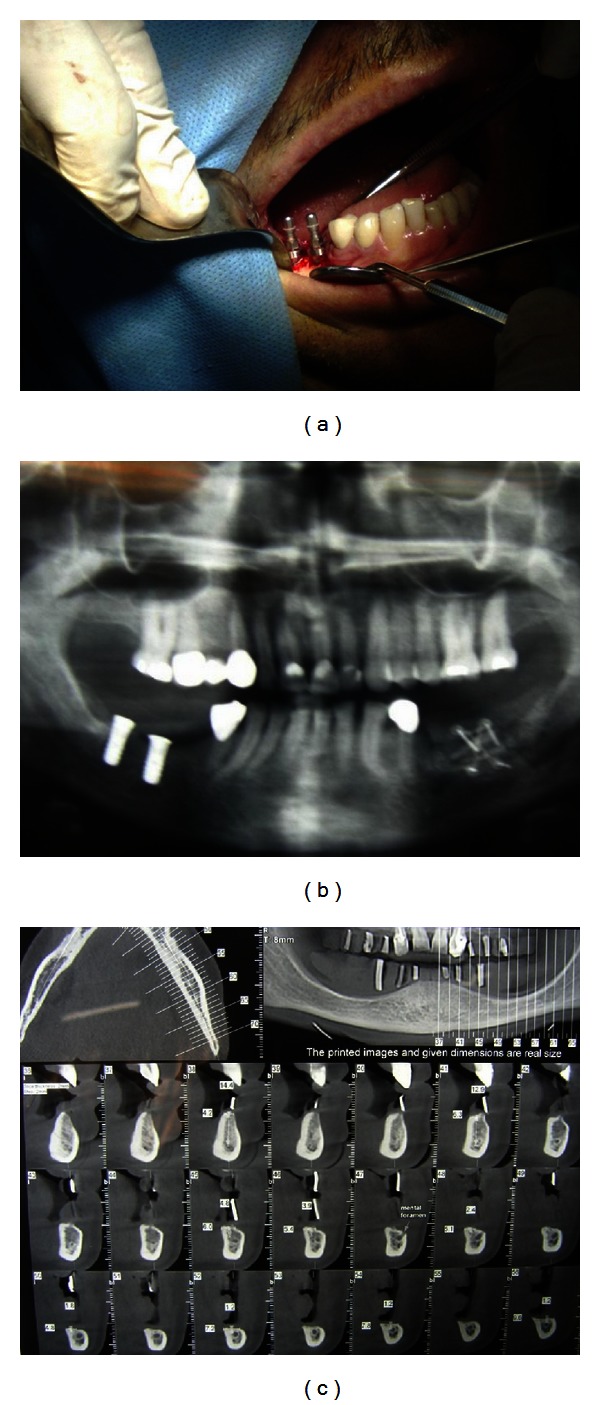
(a) Patient number one, surgery day photo. (b) Patient number one, panoramic X-ray. (c) Patient number one, CT scan.

**Figure 4:**
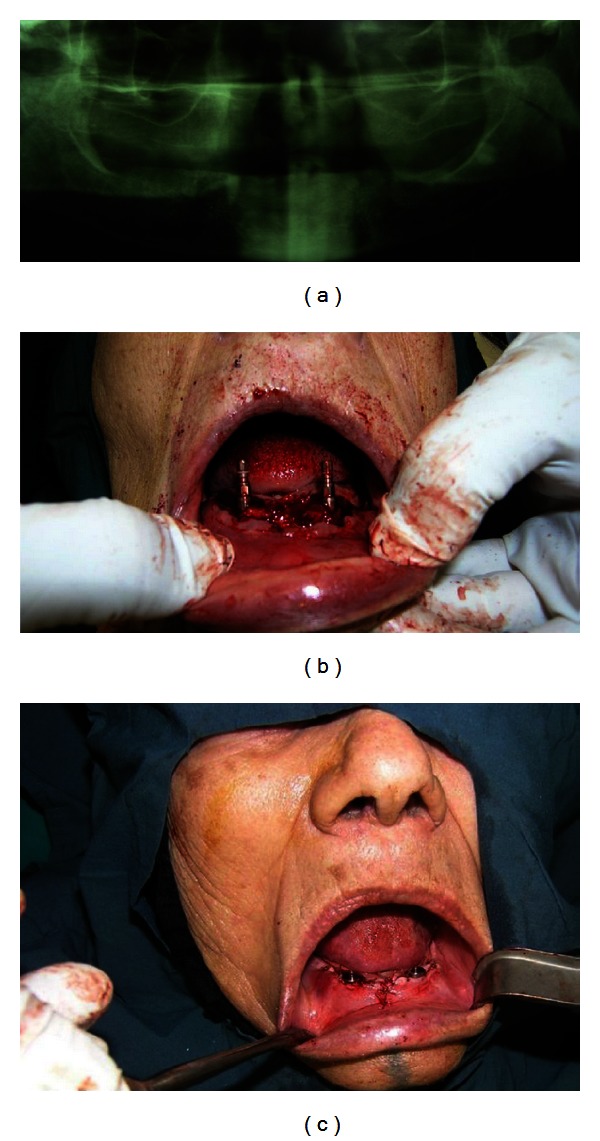
(a) Patient number two, panoramic X-ray before surgery. (b) Patient number two, surgery day photo. (c) Patient number two, after surgery photo.

Fifteen implants were examined once weekly up to five weeks and then at weeks 7 and 11 after surgery, to determine their ISQ values using Osstell mentor device. Four males and six females, 42 to 65 years old, entered the study. Table 1 summarizes the ISQ values obtained throughout the study. The mean ISQ decreases at the second measurement. Mean ISQ values continued to decrease even more significantly from the first week after placement. Starting from the forth week after surgery, however, the mean implant stability values show an increasing trend but never reach the baseline values.  Figure 2 aims to illustrate the alterations of implant stability values over time. 

## 4. Discussion

The immediate implant placement approach has been studied extensively since being introduced. Evidence available indicates that it is a successful procedure that may benefit patients. However, careful planning and case selection are needed to ensure implant success and final aesthetic outcomes [2]. There is a significant biological response by the hard and soft tissues to immediate loading of dental implants [4]. It is believed that threaded implants provide the highest mechanical stability after placement. The application of tapered implants and progressive lateral bone compression during drilling are thought to improve the implant to bone contact, implant stability, and osseointegration [5]. 

Meredith [6] and Sennerby and Meredith [7] were first to propose RFA as a highly effective qualitative method to assess implant stability. Huang et al. [8] evaluated implant behavior in different types of bones and confirmed the reliability of RFA in stability assessment. Most noticeably, authors have used RFA to trace stability alterations of implants over time. Friberg et al., [3] in a correlation analysis of the cutting torque measurements and RFA at implant placement, found satisfactory reliability of the RFA in crystal torque measurements. They also found that the stability of implants placed in softer bone seemingly raise over time with more dense bone sites; no differences in stability were observed between different bone types at week 12 which is consistent to other reports [9]. However, O'Sullivan et al. [1] compared insertion torque and bone properties in a cadaver study and obtained high values for all bone types except type IV; this was in line with the findings of Boronat López et al. [10] who reported higher ISQ values for implants inserted in areas of more compact bone. 

Other authors used RFA to determine the effects of immediate or early loading or assess changes in stability over time [3]. Resonance frequency can also be measured at any time during the process, allowing implant failure to be diagnosed at an early stage. Very low RFA values at two months appear to indicate risk of future implant failure, while ISQ values of 57–82 at one year indicate implant success [11]. RFA has been used at early stages of osseointegration and reported that ISQ values of 57–70 indicate stability. Using in vitro histomorphometric analysis, there was found no correlation between bone-implant contact (BIC) and RFA. The benefits of a rough implant surface for increased RFA-measured stability are also confirmed [12]. Authors have also compared the different locations of mandibular and maxillary ITI implants and found a significant correlation between these variables. They also observed that RFA measurements can identify unstable implants. Experimental studies used RFA to determine the stability of implants placed in irradiated bone and found that irradiation had an adverse effect on bone vascularization and hence on implant stability. It has been discussed that the objective assessment using the RFA method has made it possible to quantitatively and qualitatively analyze the stability of various types of implants and examine their behavior under different bone and loading conditions [13]. The results of the present prospective study are consistent to the fact that mandibular bone enjoys sufficient quality to render a reliable IL possible. All the implants in the present study achieved sufficient primary stability observing a one-stage surgery protocol. Systemically compromised patients were excluded from the present study to eliminate the biasing effect of conditions like osteoporosis noticed in some reports [14]. Authors have used various measures to assess qualitatively and quantitatively the implant stability. Other methods like insertion and removal torque assessment, which determine the conditions of the implant-bone interface, cannot be used, only can be used for long-term assessments [3]. RFA is a noninvasive technique that can be used repeatedly for quantitative stability measurements both intraoperatively and postoperatively. Independently of the implant system used, ISQ values obtained via RFA can be compared. Despite numerous advantages, RFA suffers from a lack of sensitivity to the quality of surrounding bone [15]. The more commonly used conventional bone-drilling was performed. Some authors have suggested superior outcomes following a bone-condensation technique where the bony walls of the implantation site are progressively condensed to compensate for a lower bone quality when needed [2]. Bone micromorphology has a prevailing effect over implant design on intraosseous initial implant stability, and it is more sensitive in terms of revealing biomechanical properties at the bone-implant interface in comparison with ISQ [15]. 

## Figures and Tables

**Table 1:** The mean (±standard deviation) of the stability values measured using resonance frequency analysis for 15 implants weekly until the 5th week and then at the 7th and 11th weeks after-placement.

Week(s)	Patients	Range	Mean ± SD	95% CI
Surgery day	15	67–85	77.20 ± 4.92	2.49
1	15	72–80	77.00 ± 3.51	1.78
2	15	64–80	74.20 ± 3.82	1.93
3	15	62–80	73.20 ± 5.19	2.62
4	15	62–79	72.13 ± 5.69	2.88
5	15	63–78	72.33 ± 4.62	2.34
7	15	66–79	73.55 ± 3.89	1.79
11	15	70–80	75.60 ± 3.72	1.88

## References

[1] O’Sullivan D, Sennerby L, Meredith N (2000). Measurements comparing the initial stability of five designs of dental implants: a human cadaver study. *Clinical Implant Dentistry and Related Research*.

[2] Koh RU, Rudek I, Wang HL (2010). Immediate implant placement: positives and negatives. *Implant Dentistry*.

[3] Friberg B, Sennerby L, Meredith N, Lekholm U (1999). A comparison between cutting torque and resonance frequency measurements of maxillary implants: a 20-month clinical study. *International Journal of Oral and Maxillofacial Surgery*.

[4] Javed F, Romanos GE (2010). The role of primary stability for successful immediate loading of dental implants. A literature review. *Journal of Dentistry*.

[5] Petrie CS, Williams JL (2005). Comparative evaluation of implant designs: influence of diameter, length, and taper on strains in the alveolar crest—a three-dimensional finite-element analysis. *Clinical Oral Implants Research*.

[6] Meredith N (1998). Assessment of implant stability as a prognostic determinant. *The International Journal of Prosthodontics*.

[7] Sennerby L, Meredith N (1998). Resonance frequency analysis: measuring implant stability and osseointegration. *Compendium of Continuing Education in Dentistry*.

[8] Huang H-M, Lee S-Y, Yeh C-Y, Lin C-T (2002). Resonance frequency assessment of dental implant stability with various bone qualities: a numerical approach. *Clinical Oral Implants Research*.

[9] Barewal RM, Oates TW, Meredith N, Cochran DL (2003). Resonance frequency measurement of implant stability in vivo on implants with a sandblasted and acid-etched surface. *International Journal of Oral and Maxillofacial Implants*.

[10] Boronat López A, Peñarrocha Diago M, Martínez Cortissoz O, Mínguez Martínez I (2006). Estudio del análisis de frecuencia de resonancia tras la colocación de 133 implantes dentales. *Medicina Oral, Patología Oral y Cirugía Bucal*.

[11] Meredith N, Shagaldi F, Alleyne D, Sennerby L, Cawley P (1997). The application of resonance frequency measurements to study the stability of titanium implants during healing in the rabbit tibia. *Clinical Oral Implants Research*.

[12] Huwiler MA, Pjetursson BE, Bosshardt DD, Salvi GE, Lang NP (2007). Resonance frequency analysis in relation to jawbone characteristics and during early healing of implant installation. *Clinical Oral Implants Research*.

[13] Chung S, McCullagh A, Irinakis T (2011). Immediate loading in the maxillary arch: evidence-based guidelines to improve success rates: a review. *Journal of Oral Implantology*.

[14] Markovic A, Calvo-Guirado JL, Lazic Z Evaluation of primary stability of self-tapping and non-self-tapping dental implants. A 12-week clinical study.

[15] Östman PO, Hellman M, Wendelhag I, Sennerby L (2006). Resonance frequency analysis measurements of implants at placement surgery. *The International Journal of Prosthodontics*.

